# A modified surgical approach for hard and soft tissue reconstruction of severe periimplantitis defects: laser-assisted periimplant defect regeneration (LAPIDER)

**DOI:** 10.1186/s40729-020-00218-6

**Published:** 2020-06-10

**Authors:** Robert Noelken, Bilal Al-Nawas

**Affiliations:** 1 Private Practice for Oral Surgery, Paradiesplatz 7-13, 88131 Lindau/Lake Constance, Germany; 2 Department of Oral and Maxillofacial Surgery, University Medical Center, Johannes Gutenberg University of Mainz, Mainz, Germany

**Keywords:** Periimplantitis, Laser, Bone grafting, Soft tissue grafting, Soft tissue Thickness, Bone regeneration, Recession, CB-CT, Attached mucosa, Ultrasonic measurement

## Abstract

**Background:**

The main problem in periimplantitis is often the combination of severe periimplant bone loss with a contaminated implant surface and an insufficient soft tissue situation. Classic surgical concepts with crestal access to the bony defect and debridement of the surface most often lead to partial defect regeneration and a soft tissue recession. An incision directly above the pathologic bony lesion is contrary to general surgical treatment rules.

**Aim:**

To overcome this problem, a new surgical concept was developed which allows to clean the implant surface, reconstruct the bony defect, and improve soft tissue height and thickness without cutting the papilla complex. This publication presents the innovative regenerative treatment approach for severe periimplantitis defects.

**Material and methods:**

After diagnosis and non-surgical pre-treatment of a severe periimplantitis lesion, the following treatment protocol was applied: horizontal mucosal incision 5 mm apical to marginal mucosa, supraperiosteal preparation in apical direction, cutting through periosteum at the level of the implant apex, subperiosteal coronal flap elevation, exploration and cleaning of the periimplant defect, thorough debridement of the implant surface with the Er:YAG laser, subperiosteal grafting with connective tissue, grafting of the bony defect with autogenous bone chips from the mandibular ramus, and bilayered suturing of periosteum and mucosa. Implant survival, marginal bone levels, periimplant probing depths, recession, and facial mucosa thickness (PIROP ultrasonic measurement) were evaluated in a pilot case at 1-year follow-up examination.

**Results:**

Inter-proximal, oral, and buccal marginal bone levels increased significantly to the level of the implant shoulder from pre-operative to 1-year follow-up examination. No signs of suppuration or periimplant infection were present. Probing depths and recession decreased significantly, while the facial mucosa thickness improved from pre-operative to final examination.

**Conclusions:**

Marginal bone levels and soft tissue improvement suggest feasibility for the regeneration of severe periimplant hard and soft tissue deficiencies by this new treatment approach. With the use of this concept, the simultaneous implant surface cleansing and improvement of hard and soft tissue seem to be possible and unfavorable postoperative exposition of titanium surface might be prevented. Comparative studies are planned to quantify the effects of this new surgical protocol.

## Introduction

Soft tissue health around dental implants, the prevention and treatment of periimplantitis, and the maintenance of periimplant esthetics have become an important topic in implant dentistry. The main problem in periimplantitis is often the combination of severe perimplant bone loss with a contaminated implant surface and an insufficient soft tissue situation.

Surgical regenerative treatment is a predictable option in managing periimplantitis and improving clinical parameters of periimplant tissues, even there wasn't found an advantage of membrane use for bone graft coverage or the submergence of the implant site following peri-implant defect treatment on periimplant regeneration [1].

Classic surgical concepts in the treatment of periodontal or periimplantitis deficiencies with at crestal access flap to the periodontal or bony defect and debridement of the root or implant surface most often lead to partial defect regeneration and a soft tissue contraction and an increase in gingival recession [2, 3]. Even when the periodontal or periimplant parameters might be improved, the reduction of soft tissue volume is especially under esthetic aspect in the esthetic zone most often unacceptable.

An incision above the pathologic bony lesion is contrary to basic surgical treatment rules. Improved flap designs by means of a microsurgical approach led to reduction of periodontal trauma and a limited morbidity [4, 5], but still did not increase soft tissue esthetics.

The use of subepithelial connective tissue for grafting of sites with periodontal deficiencies and/or mucogingival recessions at the teeth is the gold standard procedure to provide significant root coverage, clinical attachment, and keratinized tissue gain ([6], Chambrone, [7–9]). This technique is used with coronal advanced flap procedure [10, 11] or tunnel technique [12]. The use of an additional connective tissue graft seems to improve the esthetic outcome significantly.

A new technique for the treatment of teeth with advanced periodontal support loss was recently described. They used an apical incision to access the periodontal defect and prevented thereby the marginal incision (non-incised papilla surgical approach (NIPSA)) for the periodontal regeneration procedure [13]. A modification of the NIPSA combined the apical incision with the incorporation of a connective tissue graft for periodontal regeneration and the support of a composite graft of deproteinized bovine bone xenograft (Bio-Oss, Geistlich) and an enamel matrix derivative (EMD, Emdogain, Straumann) [14]. The NIPSA enables better conditions for marginal healing and soft tissue support, and the early results show significant improvements in comparison to marginal incision techniques.

**Fig. 1:**
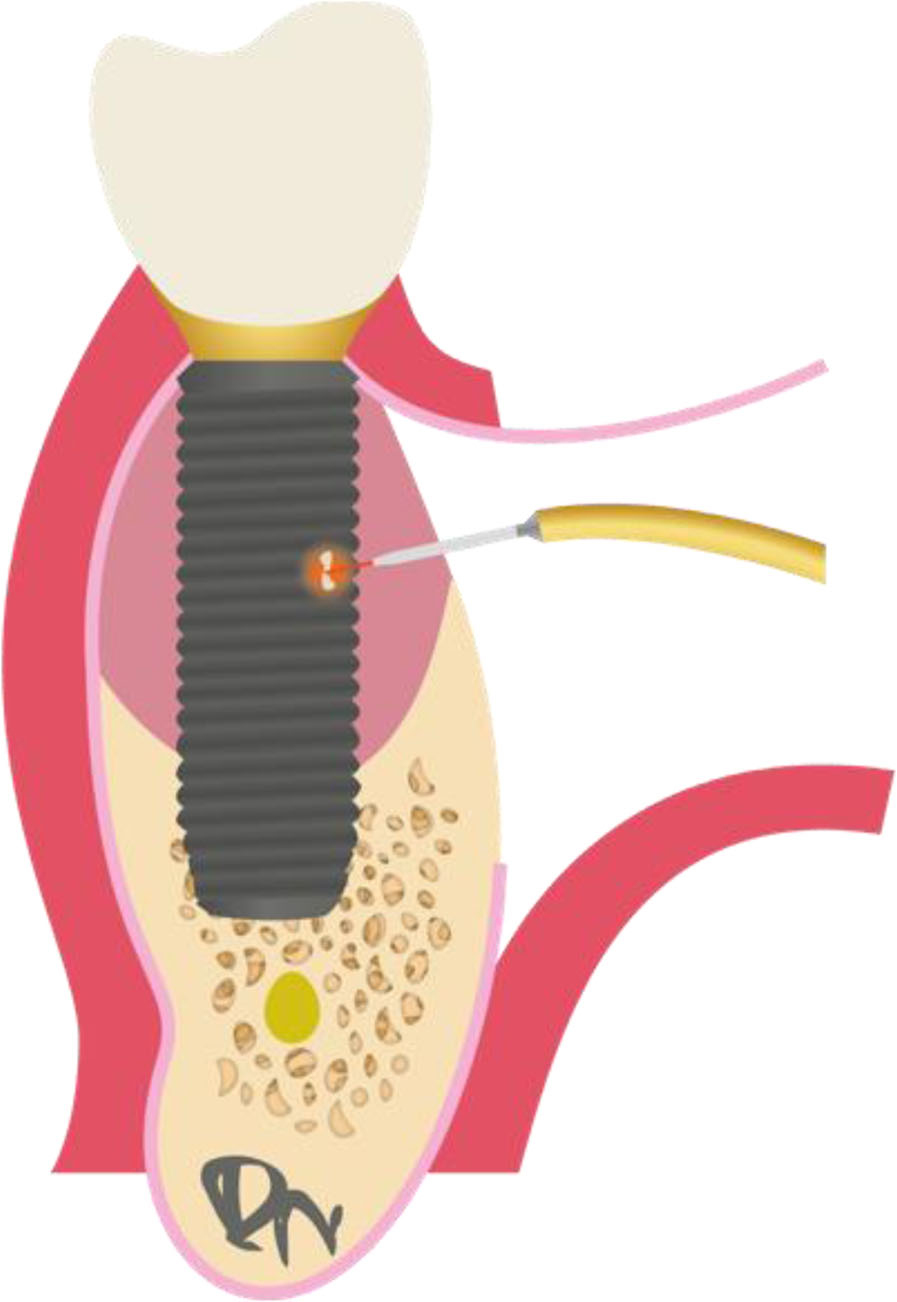
Illustration of the LAPIDER treatment concept: a periimplantitis lesion around an implant with debris on the implant surface and circumferential bone loss and soft tissue recession is cleaned by the support of the Er:YAG laser

To overcome this problem, a new surgical concept was developed which allows to decontaminate and clean the implant surface, reconstruct the bony defect, and improve soft tissue height and thickness without cutting the papilla complex (Fig. 1). This technique was invented by the first author and received the name LAPIDER for laser-assisted periimplant defect regeneration.

This case documentation evaluates the 1-year outcome of an implant with severe periimplantitis treated with this modified approach for regenerative periimplantitis therapy.

## Material and methods

### Patient

A pilot case with a severe periimplant defect was treated in November 2018 to present this new surgical approach. This technique can be used in any location of the maxilla or mandible. In the presented case the periimplant lesion presented at an implant in the left posterior mandible. The Astra Tech Profile implant was inserted to replace the first molar in a narrow crest with buccal simultaneous bone grafting 2 years before. The female patient was healthy but was smoking between 10 and 20 cigarettes a day. She interrupted smoking just 1 week following implant surgery.

### Pre-treatment examination

At pre-treatment examination, a periapical x-ray was recorded to evaluate the marginal bone level at the implant site. The periimplant probing depths were between 8 and 10 mm, the soft tissue recession was 4.5 mm, and the width of the keratinized mucosa was 21.5 mm. According to the classification of Schwarz [15], the periimplantitis defect was classified as class Id. The bone loss was circumferential with a deep dehiscence bone loss on the buccal aspect. Bone loss was 9 mm at the buccal and 6 mm at the oral aspect apical to implant shoulder. The gingival biotype was determined as a thin tissue biotype [16].

### Surgical technique

**Fig. 2:**
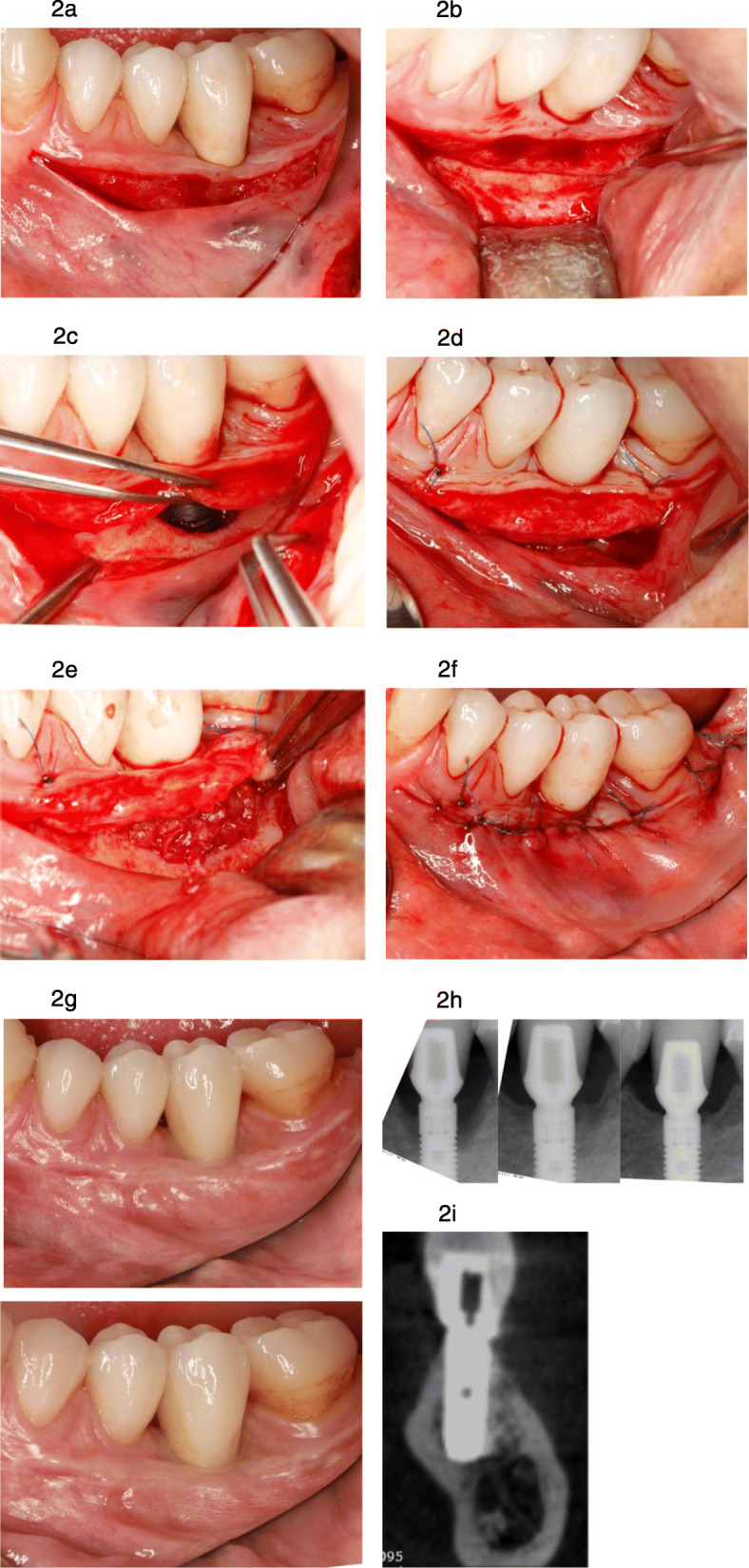
Treatment of a severe perimplantitis lesion at implant in the posterior left mandible according to LAPIDER technique.**a**Horizontal mucosal incision and split-flap preparation in apical direction.**b**Separation of the periosteum at the level of the implant apex.**c**Subperiosteal coronal flap elevation and exploration and cleansing of the periimplant defect and the implant surface with the Er:YAG laser.**d**Subperiosteal grafting with connective tissue, which was harvested at the palate.**e**Augmentation of the bony defect with autogenous bone chips from the mandibular ramus.**f**Covering the grafted site by reflecting and suturing periosteum to periosteum. Wound closure is completed by mucosal suturing.**g**At 6-month and 1-year follow-up examination, the signs of a periimplant infection are gone while the soft tissue level and thickness at the implant site are improved.**h**Periapical X-rays pre-operatively at 6-month and at 1-year follow-up show the complete regeneration of the severe bony defect to the level of the implant shoulder.**i**CB-CT at 6 months reveals the bony regeneration to the level of the implant shoulder at the oral and the buccal aspects

The implant was treated according to the LAPIDER treatment protocol: horizontal mucosal incision 4 to 5 mm apical to marginal mucosa, supraperiosteal split-flap preparation in apical direction (Fig. 2a), cutting through the periosteum at the level of the implant apex (Fig. 2b), subperiosteal coronal flap elevation, exploration and cleaning of the periimplant defect using a micro-surgical approach under a chair-side microscope (Fig. 2c), thorough debridement of the implant surface with the Er:YAG laser (AdvErL EVO, J. Morita Europe, Dietzenbach, Germany), and subperiosteal grafting with connective tissue (Fig. 2d). Simultaneous periimplant defect grafting was performed by condensing autogenous bone chips to the bottom of the defect for reconstruction of the periimplant defect (Fig. 2e). Autogenous bone grafts were harvested by a bone scraper (Micross, Meta, Reggio Emilia, Italy) at the mandibular ramus. The periosteum was reflected apically to cover the graft and sutured to the periosteum by a monofilamentous resorbable suture (Monocryl 5-0, Ethicon, Norderstedt, Germany). The bilayered suturing was finished with mucosal adaptation by a non-resorbable suture (Prolene 6-0, Ethicon, Norderstedt, Germany) (Fig. 2f).

The implant surface was irridated using the PS600 tip (J. Morita) on the Er:YAG laser. The flat quartz tip tapers from 600 μm in the upper portion to 400 μm in the lower portion. The distance from the end of the tip to the implant surface should be very close but without direct contact. Surface ablation was set to 50 mJ/mm^2^, and the pulse was set to 20 pulses per second. Sterile water was injected at a rate of 5 ml/min. Irridation time to clean the contaminated implant surface precisely was about 7 min. Access to the lingual implant surface was provided by entering the defect through the lingual pocket with the slim laser tip under visual control from the buccal aspect.

### Follow-up and definition of outcome parameters

The patient was examined preoperatively, at the time of periimplantitis surgery, at 3 and 6 months, and at 1-year following periimplantitis treatment (Fig. 2g).

### Interproximal marginal bone level

The status of the interproximal marginal bone level was assessed using digital periapical radiographs (Fig. 2h).

### Buccal bone level and thickness

The thickness of the facial bone wall was determined by CB-CT data, specifically by the reconstruction according to the long axis of the implant at follow-up examination (Fig. 2i). The thickness of the facial bone wall was measured 1 mm, 3 mm, and 6 mm apical to the buccal implant shoulder level [17, 18].

### Probing depths

The periimplant probing depths were measured at 6 sites around the implant by a periodontal probe with 1 mm calibration.

### Width of keratinized mucosa

The width of the keratinized and attached mucosa at the midfacial aspect of the implant site was measured by a periodontal probe with 1 mm calibration.

### Soft tissue recession

The midfacial soft tissue recession was calculated in relation to a tangent between the CEJs of the neighboring teeth.

### Buccal mucosa thickness

The facial mucosa thickness was measured using an ultrasonic device with 20 MHz frequency and a 1540 m/s ultrasonic impulse velocity (PIROP Biometric Scanner; Echoson, Pulawy, Poland). The measurements were performed at a level 4 mm apical to the midfacial mucosal margin at the implant. Minimal pressure was applied to avoid compression of the mucosa.

## Results

### Implant follow-up

At 1-year follow-up, the implant was in function without any signs of infection.

### Interproximal marginal bone level

The interproximal bone level changed from − 5.5 mm below to the level of the implant shoulder.

### Buccal bone level and thickness

The buccal bone loss of 9 mm (measured clinically at periimplantitis surgery) regenerated to the level of the buccal implant shoulder (measured by CB-CT, Fig. 2i). The thickness of the buccal bone wall was 0.6 mm at 1 mm, 1.3 mm at 3 mm, and 3.1 mm at 6 mm.

### Probing depths

The periimplant probing depths decreased from 8 to 10 mm to 2 to 3.5 mm.

### Width of keratinized mucosa

The width of the keratinized and attached mucosa at the midfacial aspect of the implant site improved from 1.5 to 3.5 mm.

### Soft tissue recession

The midfacial soft tissue recession decreased from 4.5 mm to 3 mm.

### Buccal mucosa thickness

The buccal mucosa thickness increased from 1.53 mm at pre-operative examination to 2.42 mm at 1-year follow-up.

## Discussion

The role of keratinized tissues around implants is not finally elucidated, but seems to support periimplant health even it is not necessary in sites with perfect hygiene [19]. For the prevention of periimplantitis lesions, a systematic review [20] highlighted the clinical relevance of keratinized mucosa around dental implants in preventing periimplant disease. A sufficient width of keratinized mucosa led to less mucosal inflammation, less plaque accumulation, increased stability of the periimplant area, and prevention of mucosal recession.

The additional use of connective tissue grafts in the treatment procedure of periimplantitis lesions to improve the dimensions of the keratinized tissues is described in the literature as a beneficial additional method.

To improve the benefit on wound healing and flap stability with a coronally advanced flap for the treatment of gingival recessions, a clinical study observed the inclusion of periosteum in the flap versus a split-thickness approach showing that the inclusion of the periosteum led to a significant higher rate of complete root coverage [21].

In this study, it was possible to observe a significant increase in the width of the keratinized mucosa by grafting the sites with autogenous connective tissue grafts from the palate. This is supported by studies, which presented the impact of the regeneration of keratinized attached mucosa [22] and the advantage of autogenous soft tissue grafts in comparison to xenogeneic collagen matrices with a bilaminate structure especially in sites with an initially compromized width of keratinized mucosa smaller than 2 mm [23].

A retrospective study of our workgroup analyzed the relationship between hard and soft tissue thickness and several possible impacting factors like soft tissue grafting, implant angulation, and positioning at immediately provisionalized implants [24]. The statistical analysis showed no significant correlation between facial mucosa or bone thickness and the orofacial angulation and positioning of the implants. However, an increased thickness of the facial mucosa was observed in patients with a thick gingival morphotype and in cases with simultaneous connective tissue grafting. The amount of improvement of the thickness of the facial bony wall was dependent on the pre-operative deficiency. This implies, that a severe defect allows for a significant bigger amount of regeneration by immediate and flapless reconstruction with autogenous bone. This seems to be in line with the presented treatment protocol using autogenous bone and connective tissue grafts in severe periimplant defects. The data of the case report by Moreno Rodriguez et al. (Moreno Rodriguez, Ortiz Ruiz et al. 2019) show that an apical incision with Bio-Oss and EMD grafting combined with additional grafting of a connective tissue lead especially in compromised periodontal cases to an improvement in marginal tissues with reduction of pocket probing depths and gains in clinical attachment level.

The effectiveness of the cleansing procedure of the implant surface seems to have an important role on the capacity of bony regeneration around implants treated for periimplantitis. The methods of powder-abrasive therapy and the use of a Er:YAG laser seems to be the most effective procedures in non-surgical periimplantitis therapy [25]. A study using Er:YAG laser for periimplantitis treatment led to a significant reduction of probing depth and radiographically proven bone regeneration [26]. A new approach for surgical regenerative therapy of periimplantitis lesions using an electrolytic method to remove biofilms from contaminated implant surfaces was described by [27]). This technique requires the removal of the prosthetic restoration and the soft tissue coverage of the grafted site for a submerged healing. They showed that the electrolytic cleaning methods as a single procedure is effective and allows for significant clinical bone fill or complete re-osseointegration following covered defect grafting.

A present systematic review by Tomasi [28] aimed at evaluating the efficacy of reconstructive surgical therapy at periimplantitis-related bone defects. They reported a larger improvement in marginal bone levels and in defect fill for the test procedures compared to control procedures, but no differences for probing depth and bleeding on probing were found. Changes of clinical attachment and soft tissue levels were not considered. The available evidence on reconstructive therapy at periimplantitis-related defects is limited and the interpretation of the marginal bone level gain for test procedures is difficult as graft material may not be distinguishable from the newly formed bone.

The review of Madi et al. [29] considered possible surgical treatment modalities for periimplantitis defects to regain re-osseointegration. Various implant surface decontamination techniques have been used either alone or simultaneously with/without guided bone regeneration. Despite the access flap surgery, it was observed that the application of single decontamination measure either chemical or mechanical was not adequate to provide a better treatment outcome. Er: YAG laser showed no implant surface alteration and provided favorable environment for re-osseointegration.

The access to large periimplant defects at the lingual aspect is complicated and limits this technique. The slim laser tip allows to enter the bone defect and the implant surface through the lingual pocket. Simultaneous visual control from the buccal aspect is possible, but needs magnification and light. The waiver of a lingual incision is advantageous for bone regeneration by keeping the lingual tissue integrity and the maintenance of blood supply.

Even the initial results of this new approach for periimplantitis surgery are very promising, prospective studies are needed to examine the impact of this new approach of periimplantitis treatment strategy on hard and soft tissue regeneration.

## Conclusion

Marginal bone levels and soft tissue results suggest feasibility for the regeneration of severe periimplant hard and soft tissue deficiencies by this new treatment approach. With the use of this concept, the simultaneous implant surface cleansing and improvement of hard and soft tissue seem to be possible and unfavorable postoperative exposition of titanium surface might be prevented. Adding a connective tissue graft simultaneously to periimplantitis treatment did increase the vestibular soft tissue thickness in short-term observation. As a next step, a comparative study has to identify the detailed and long-term results of the technique.

## Abbreviations

CB-CT: Cone beam computed tomography
NIPSA: Non-incised papilla surgical approach
EMD: Enamel matrix derivative (Emdogain)
